# The Validation of an Analytical Method for Sulfentrazone Residue Determination in Soil Using Liquid Chromatography and a Comparison of Chromatographic Sensitivity to Millet as a Bioindicator Species

**DOI:** 10.3390/molecules190810982

**Published:** 2014-07-28

**Authors:** Marcelo Antonio de Oliveira, Fábio Ribeiro Pires, Mariana Ferraço, Alessandra Ferreira Belo

**Affiliations:** Centro Universitário Norte do Espírito Santo, UFES, Rodovia BR 101 Norte, km 60, São Mateus, ES 29932-540, Brazil; E-Mails: pires.fr@gmail.com (F.R.P.); marianaferraco@yahoo.com.br (M.F.); ferreiragro@yahoo.com.br (A.F.B.)

**Keywords:** herbicide, HPLC, *Canavalia ensiformis*, *Crotalaria juncea*, *Pennisetum glaucum*, bioindicator

## Abstract

Commonly used herbicides, such as sulfentrazone, pose the risk of soil contamination due to their persistence, bioaccumulation and toxicity. Phytoremediation by green manure species has been tested using biomarkers, but analytical data are now required to confirm the extraction of sulfentrazone from soil. Thus, the present work was carried out to analyze sulfentrazone residues in soil based on liquid chromatography with a comparison of these values to the sensitivity of the bioindicator *Pennisetum glaucum*. The soil samples were obtained after cultivation of *Crotalaria juncea* and *Canavalia ensiformis* at four seeding densities and with three doses of sulfentrazone. The seedlings were collected into pots, at two different depths, after 75 days of phytoremediator sowing and then were used to determine the herbicide persistence in the soil. A bioassay with *P. glaucum* was carried out in the same pot. High-performance liquid chromatography (HPLC), using UV-diode array detection (HPLC/UV-DAD), was used to determine the herbicide residues. The HPLC determination was optimized and validated according to the parameters of precision, accuracy, linearity, limit of detection and quantification, robustness and specificity. The bioindicator *P. glaucum* was more sensitive to sulfentrazone than residue determination by HPLC. Changes in sulfentrazone concentration caused by green manure phytoremediation were accurately identified by the bioindicator. However, a true correlation between the size of the species and the analyte content was not identified.

## 1. Introduction

Commonly used herbicide molecules, such as N-[2,4-dichloro-5-[4-(difluoromethyl)-4,5-dihydro-3-methyl-5-oxo-1H-1,2,4-triazol-1-yl]-phenyl]methanesulfonamide (sulfentrazone, SUL), pose the risk of soil contamination due to their persistence, bioaccumulation and toxicity. Herbicides eventually cause damage to subsequent crops, flora and soil fauna, in either direct or indirect ways [1,2]. Herbicide molecules begin the processes of redistribution and degradation as soon as they reach the ground. These processes can be extremely short, e.g., some simple and non-persistent molecules (2,4-D, fluazifop-*p*-butyl, clomazone, and others), or can last for months or years, such as the processes that occur with highly persistent compounds (tebuthiuron, fomesafen, and others) [3].

Sulfentrazone is recommended for pre-emergence use in weed control in sugarcane, soybean, citrus, coffee and eucalyptus plantations, as well as in non-agricultural areas, however, sulfentrazone is also among the group of herbicides that exhibits long residual periods in the soil [4]. Because sulfentrazone exhibits significant persistence in soil, this herbicide may prevent the cultivation of sensitive plants for long periods after application, depending on the dose used and the local climate conditions [5].

Among the green manure species capable of phytoremediating SUL are *Crotalaria juncea* and *Canavalia ensiformis* [6]. After planting the phytoremediation species, SUL residues can be determined by using high-performance liquid chromatography (HPLC), as well as by using the bioindicator *Pennisetum glaucum* (millet), which can be used to indicate the presence of SUL because its growth is inversely proportional to the residual amount of the herbicide.

SUL is a bronze-colored powder with a slight sulfur odor, a melting point between 120 and 122 °C, a density of 0.53 g·mL^−1^, a pKa of 6.56 and an octanol/water partition coefficient of 9.8 [7]. The water solubility of SUL varies with pH, being 110, 780 and 1600 mg·L^−1^ at pH 6.0, 7.0 and 7.5, respectively [7]. The determination of SUL residues in agronomic samples has been studied by several authors [8,9]. Chromatographic studies aim to evaluate the chemical aspects of sample extraction and the possibility of the formation of degradation products. Furthermore, it is important to compare the sensitivity of the chromatographic method with that of a method using the bioindicator *Pennisetum glaucum*, followed by establishing correlations.

To ensure the reliability of the analytical results, the methodology used to dose the SUL must be validated. An analytical method is considered officially validated when the associated parameters, such as precision, accuracy, linearity, limit of detection and quantitation, specificity and robustness, are adequate [10]. The specificity can be assessed from the analyte when the analyte is subjected to stress conditions, also known as the intrinsic or inherent stability. Stress conditions include the effects of temperature, moisture, oxidation, exposure to light and hydrolysis at different pH values [11].

The reliability of a chromatographic analytical method is also determined by the suitability parameters of the system, known as the “system suitability” or “performance parameters.” The main parameters evaluated are the retention factor (k’), number of theoretical plates (N), resolution (R) and tailing or asymmetry factor (T) [10,12,13,14].

The retention factor (k') assesses the degree of analyte affinity for the stationary phase. The optimal value of k′ must be situated between 0.5 and 20, or must be greater than 2, in accordance with [13,14]. The number of theoretical plates (N) estimates the efficiency of the column in analyte separation. It is reported that the number of theoretical plates per column must be at least 2000 [10,14,15]. The resolution (Rs) parameter quantifies the degree of separation between two substances and should be greater than 1.5 or greater than 2.0, in accordance with [13,15]. The tailing factor (T) evaluates the peak symmetry and has a value of 1 when the peak is perfectly symmetric. As T increases, the accuracy of the analysis becomes less reliable [12]. Some authors report that values lower or equal to 2 are acceptable [14,15].

Considering the persistence of sulfentrazone molecules in soil, it is essential to assess its residues with a validated analytical method, such as HPLC, and to compare the sensitivity of the chromatographic method with the sensitivity of the bioindicator *Pennisetum glaucum*. Thus, the present work was carried out with the objectives of evaluating sulfentrazone residues in the soil using liquid chromatography and comparing the results to the sensitivity of the bioindicator *Pennisetum glaucum*.

## 2. Results and Discussion

### 2.1. Validation of the HPLC Methodology

The analytical methodology proposed for the analysis of SUL was optimized and validated with a mobile phase consisting of acetonitrile—phosphoric acid 0.1% (60:40), a 5 µm RP-18 Sunfire^®^ column measuring 250 mm × 4.6 mm with a RP-18 Sunfire^®^ pre-column, a flow rate of 1 mL·min^−1^, an injection size of 10 µL with detection at 220 nm, a column temperature of 30 °C.

The precision in the quantification of sulfentrazone showed a RSD (%) lower than 2%, for both the repeatability and the intermediate precision measurements. The intra-day and inter-day accuracies, which refer to the percentage of recovery of the analyte, were 102.42% and 102.83%, respectively.

The linearity was verified in the range of 0.175 and 0.325 µg·mL^−1^ of soil matrix. The value of the linear correlation coefficient (r) was 0.9993, which is satisfactory, and the following linear equation was obtained: y = 29999.94311x + 130.14757. The limits of detection and quantification were determined for SUL in the soil samples; the values were 8.7699 ng·mL^−1^ and 26.5754 ng·mL^−1^, respectively. They were evaluated according the three linearity curves.

**Figure 1:**
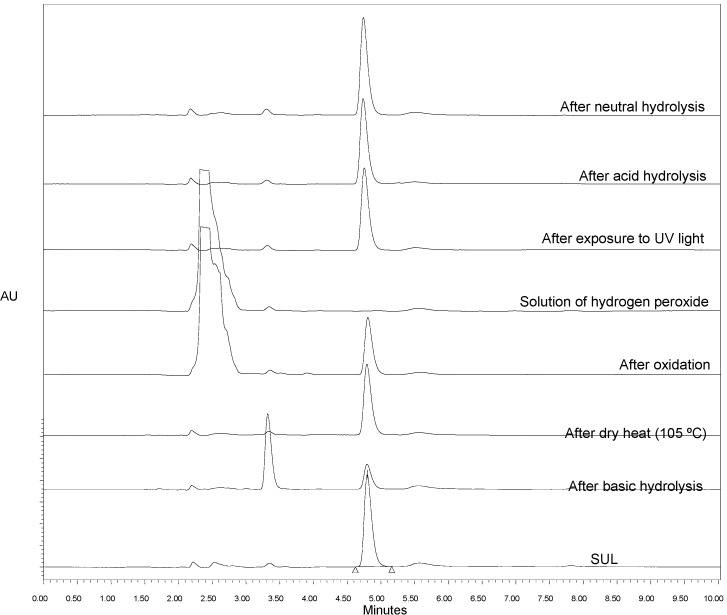
Chromatograms of sulfentrazone (SUL) before (standard) and after subjection to stress by exposure to dry heat, exposure to UV light, oxidation, neutral hydrolysis, acid and basic hydrolyses and hydrogen peroxide solution.

The specificity was measured in the presence of degradation products after the analyte was subjected to stress conditions. Figure 1 shows the chromatograms of SUL (standard) and the samples after being subjected to the stress conditions, which included neutral, acid and basic hydrolyses, oxidation, exposure to dry heat and exposure to UV light. Additionally, the chromatogram of the hydrogen peroxide solution is also presented.

The results from Figure 1 show that SUL has a retention time (t_R_) of 4.8 min. Moreover, the sample that exhibited degradation was subjected to a basic hydrolysis stress, from which a degradation product (DP1) was formed with a retention time of 3.2 min. It is also worth highlighting that the peak at a t_R_ (retention time) of 2.4 min of the sample subjected to oxidation corresponds to the peak of hydrogen peroxide, as seen in the chromatogram resulting from the analysis of the hydrogen peroxide solution.

### 2.2. System Suitability

**Figure 2:**
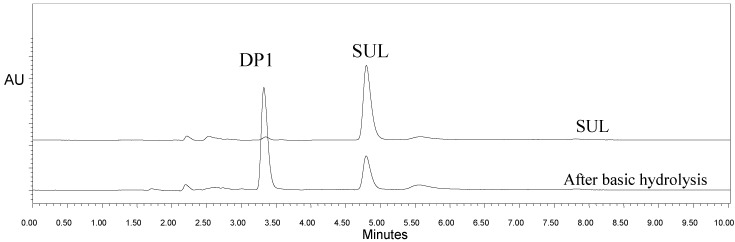
Chromatograms of sulfentrazone (SUL) before (Standard) and after being subjected to stress by basic hydrolysis, emphasizing product formation by cleavage of the lactone ring (DP1).

The specificity was adequate, and the resolution (Rs) between the peaks was satisfactory. The resolution between SUL (t_R_ = 4.8 min) and DP1 (t_R_ = 3.2 min) was 7.96, indicating a good separation between the peaks, as seen in Figure 2.

**Figure 3:**
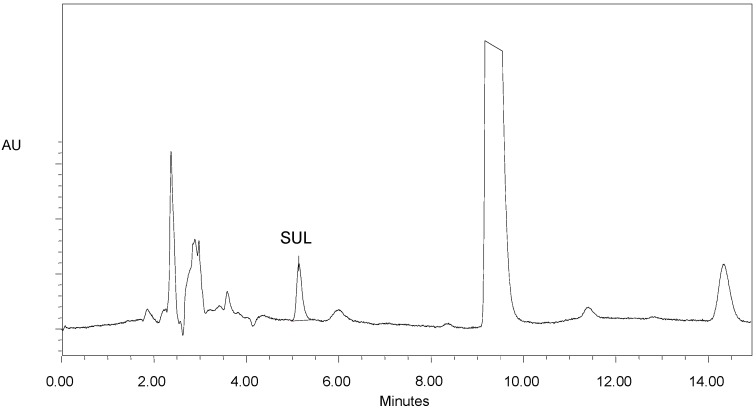
Chromatogram of a soil sample contaminated with sulfentrazone (SUL), highlighting the peak of the analyte.

The specificity was also evaluated relative to the matrix, *i.e.*, the soil. Figure 3 shows that the separation of the analyte (SUL) relative to the contaminants of the matrix was good, which demonstrates the specificity of the system. It is worth noting that the validation was carried out using the matrix as the reference and that the time of analysis (chromatographic run) was 15 min exactly because a contaminant with an approximate t_R_ of 14 min was observed.

**Figure 4:**
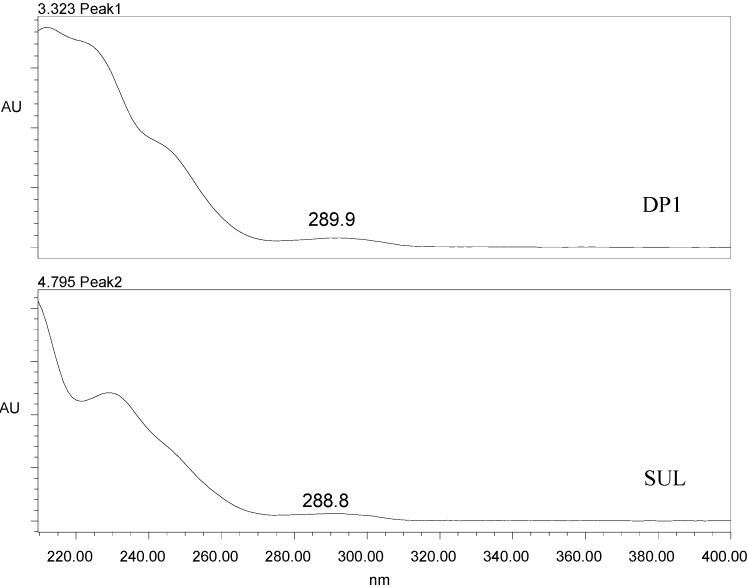
Spectra obtained using the UV/DAD detector in the range from 200 to 400 nm for the peaks relative to the product formed by cleavage of the lactone ring (DP1) and sulfentrazone (SUL).

The peak purity measurement is a specificity requirement. According to the instruction manual of the Waters equipment, to be considered pure, a peak must have a Purity Angle < Purity Threshold. The peak of SUL showed a Purity Angle of 0.186 and a Purity Threshold of 0.361 in the presence of the degradation product obtained by basic hydrolysis and within the soil samples. This demonstrates that SUL does not co-elute with any other substance. Figure 4 shows the spectra obtained with the UV/DAD detector for the SUL (t_R_ = 4.8 min) and DP1 (t_R_ = 3.2 min) peaks.

**Scheme 1:**
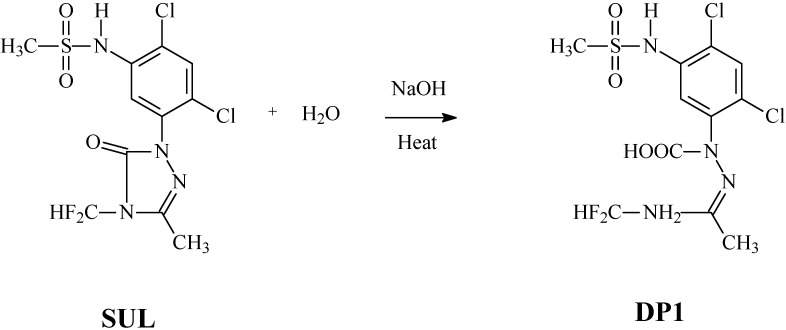
The reaction scheme of the basic hydrolysis of sulfentrazone (SUL), giving rise to DP1, which is the product formed by cleavage of the lactone ring.

Because the spectra of SUL and DP1 are quite similar, we suggest that DP1 is formed by the cleavage of the lactone ring through the basic hydrolysis of SUL, as depicted in the Scheme 1. Most of the chromophoric component of the SUL molecule is maintained in DP1, which is in agreement with the similarity observed between the spectra. Additionally, DP1 has a more polar structure than SUL, which explains its lower retention time in reverse phase chromatography.

The suitability of the chromatographic system was evaluated for the SUL peak. The retention factor (k') was equal to 1.30; the peak asymmetry (W_0.5%_) was satisfactory with a value of 1.35; and the number of theoretical plates (N) was equal to 7886 plates/column. The robustness was assessed by changing three analytical conditions. The flow rate was altered to 2 mL·min^−1^; the temperature was varied to 40 °C; and the mobile fraction was changed by increasing the fraction of acetonitrile to 65%. The results were satisfactory for these parameters.

### 2.3. Residual Evaluation of SUL in the Soil Using the Bioindicator Pennisetum glaucum

Table 1 compiles the results for the height (cm) measurements collected at 25 and 42 days after sowing the *Pennisetum glaucum* (millet) plants, which were cultivated after the cultivation of *C. ensiformis* in the soils contaminated with two different doses of the SUL herbicide. The measurements extracted from the contaminant-free soil are also presented. After 25 and 42 days, the height of the millet was reduced with increasing SUL dose. When the *C. ensiformis* plants were not cultivated prior to the millet and a dose of 400 g·ha^−1^ of SUL was applied, the *P. glaucum* was unable to survive. This finding can be explained by the absence of phytoremediation, which led to the consequent persistence of SUL (Table 1). This demonstrates the phytoremediator effect of *C. ensiformis* when preceding millet and also emphasizes the importance of phytoremediation in the decontamination of soils contaminated with SUL. For this same species, some authors found similar results for the phytoremediation of the herbicides tebuthiuron and trifloxysulfuron sodium, respectively [16,17].

The prior cultivation of *C. ensiformis* leads to increased *P. glaucum* plant heights after 25 and 42 days, for both doses, *i.e.*, 200 g·ha^−1^ and 400 g·ha^−1^ of SUL, as shown in Table 1. This demonstrates the sensitivity of the bioindicator for detecting the reduction of the SUL concentration in the soil through phytoremediation.

**Table 1:** The height of *Pennisetum glaucum* at 25 and 42 days, sown after the prior cultivation of *Canavalia ensiformis* and *Crotalaria juncea* in soil contaminated with two doses of the herbicide sulfentrazone.

**Sulfentrazone Doses (g·ha^−1^)**	***C. ensiformis* Plants m^−2^**			
	0	10	0	10
	**Height (cm) after 25 Days**		**Height (cm) after 42 Days**	
0	20.37 Aa	19.93 Aa	114.65 Aa	124.87 Aa
200	11.96 Bb	15.22 Ab	67.42 Bb	80.37 Ab
400	0.00 Bc	7.02 Ac	0.00 Bc	21.62 Ac
RSD (%)	15.05		12.29	
**Sulfentrazone Doses (g·ha^−1^)**	***C. juncea* Plants m^−2^**			
	0	60	0	60
0	18.32 Aa	18.52 Aa	100.50 Aa	101.93 Aa
200	12.76 Ab	13.93 Aab	58.37 Ab	76.87 Aa
400	0.00 Bc	8.92 Ab	0.00 Bc	35.00 Ab
RSD (%)	23.59		22.96	

* Means followed by the same lower case letter in the column and capital letter in the line, on the same date, do not differ by the Tukey test at 5%. RSD: relative standard deviation.

This demonstrates the sensitivity of *P. glaucum* toward small changes in the concentration of sulfentrazone. Others have already reported a high sensitivity of *P. glaucum* to SUL, where the residual activity of the herbicide it was analyzed [18]. Thus, it can be seen that small changes in SUL concentration in the soil following phytoremediation were detectable during millet cultivation. However, this is only a comparative result, and it is not possible to consider this finding as an analytical result of persistent residual concentration in the soil. From a practical standpoint, the use of the bioassay is very interesting due to its simplicity, practicality and the reliability of knowing that, agronomically, residues of SUL exist at phytotoxic levels in soil. Such information can guide the management in terms of all safety concerns in areas that receive applications of sulfentrazone.

Additionally, Table 1 shows the results for the height (cm) at 25 and 42 days after sowing the *P. glaucum* plants cultivated after the prior cultivation of *C. juncea* in soil that was also contaminated with two doses of SUL herbicide. After 25 and 42 days, the height of the millet decreased with increasing SUL dose. It was also found that when the herbicide was applied to the soil at a higher dose (400 g·ha^−1^), *P. glaucum* did not develop due to the high toxicity of the herbicide. This demonstrates the significant phytoremediation effect of *C. juncea*. Plant height is a useful reference in phytoremediation studies [19], indicating an effective approach to studying the remediating role of various species of green manure [20].

The prior cultivation of *C. juncea* also leads to an increase in the *P. glaucum* plant heights after 25 and 42 days at SUL doses of 200 g·ha^−1^ and of 400 g·ha^−1^, as shown in Table 1. This demonstrates the sensitivity of the bioindicator for detecting reduced SUL concentrations in the soil, where the reduction is provided by the phytoremediation effect of *C. juncea*, as it was observed with *C. ensiformis*. This also shows the high sensitivity of millet for the detection of changes in residual SUL concentration in the soil.

### 2.4. Residual Evaluation of SUL in the Soil by HPLC and a Comparison of the Sensitivity of the Two Methods

Table 2 shows the amounts of residual SUL found at two depths (0.0 to 0.10 and 0.10 to 0.20 m) after the prior cultivation of *C. ensiformis* and *C. juncea* for comparison based on the sensitivity of the bioindicator. The percent of SUL that was recovered in the soil relative to the total applied amount shows that the remaining fraction after phytoremediator cultivation was low. The herbicide can have various destinations after reaching the soil, including fixation/adsorption by the soil, plant uptake, and/or physico-chemical-biological degradation, depending on the characteristics of the molecule itself and on the local environmental conditions. In phytoremediation, it is expected that organic molecules will be absorbed and degraded to nontoxic compounds [21,22].

**Table 2:** Amount of sulfentrazone found at two depths of soil contaminated with two doses of herbicide, after the prior cultivation of *Canavalia ensiformis* and *Crotalaria juncea.*

Sulfentrazone Doses (g·ha^−1^)		*C. ensiformis* m^−2^				***C. juncea* m^−2^**			
		0		10		0		60	
		**µg of Sulfentrazone per g of Soil**							
0.0–0.10 (m)	0	0.0000 Ac	-	0.0000Ab	-	0.0000 Ac	-	0.0000Ac	-
	200	0.0183 Ab	(9.89) *	0.0148 Ba	(8.00)	0.0163 Ab	(8.81)	0.0160 Ab	(8.64)
	400	0.0297 Aa	(16.05)	0.0138 Ba	(7.45)	0.0222 Aa	(12.00)	0.0210 Ba	(11.35)
RSD (%)		11.48				3.24			
0.10–0.20 (m)	0	0.0000 Ac	-	0.0000Ab	-	0.0000Ac	-	0.0000Aa	-
	200	0.0138 Ab	(7.45)	0.0100 Ba	(5.40)	0.0146 Ab	(7.89)	0.0000Ba	-
	400	0.0285 Aa	(15.40)	0.0000Bb	-	0.0238 Aa	(12.86)	0.0000Ba	-
RSD (%)		3.69				4.28			

* Percent value quantified relative to the amount applied to the soil. Means followed by the same lower case letter in the column and capital letter in the line, on the same species, do not differ by the Tukey test at 5%. RSD: relative standard deviation.

The fractions found in soils treated by phytoremediation are always lower than the values detected in non-phytoremediated soils, which demonstrates the remediation effect of the plants. A similar result has already been observed, where it was found the ability to remediation of these same species using a bioassay technique [6]. However, the difference between the results is small, in particular, for *C. juncea*. This result also leads to the conclusion that *C. ensiformis* is slightly more efficient than *C. juncea* in the phytoremediation of soils contaminated with sulfentrazone at the depth of 0.0 to 0.10 m, however the opposite occurred at 0.10 to 0.20 m.

It is also worth noting that the herbicide sulfentrazone is retained in higher amounts at the superficial layer of the soil, in comparison with the lower SUL contents recorded at the depth of 0.10 to 0.20 m. However, this finding can also be attributed to the likely higher absorption of the herbicide at the depths at which the roots accumulate in higher volumes (0.10 to 0.20 m). In this case, it can be concluded that the sulfentrazone was not detected due to the increased efficiency of phytoremediation at greater depths.

Table 2 shows that even if the soil was contaminated by SUL, it was not possible to detect and quantify the analyte. However, SUL was accessed and absorbed by the bioindicator plant, even at levels lower than those detected by the chromatography analysis, as was evidenced by the symptoms of intoxication exhibited by the plants, which resulted in reduced *P. glaucum* plant heights (Table 1). Therefore, for investigations involving the carryover effect of residues in the soil, the bioassay correlation (biological indicator) of the intoxication behavior or tolerance to the herbicide gives a more precise indication than the analytical determination [23], especially for analytes applied in doses expressed in g or mg·ha^−1^. A typical example is trifloxysulfuron sodium, which is commonly applied with an average dose of 7.5 g·ha^−1^ [20].

The bioassay methodology has been considered adequate for the detection of sulfentrazone herbicide residues in soil [24]. The work carried out by Rodrigues *et al.* showed that even if only trace sulfentrazone is found relative to the total amount applied to the soil, this amount is sufficiently large to successfully control *B. plantaginea* and to cause clear toxicity symptoms in soybean, in the field, and to forage sorghum in the bioassays [25]. A similar result was obtained by Pires *et al.* when working with picloram, which was found to be phytotoxic to soybeans grown on straw with phytoremediator species, despite the fact that the analyte was not detectable by chromatographic analysis [26].

## 3. Experimental Section

### 3.1. Validation of the HPLC Methodology

The analytical method proposed for the analysis of SUL was based on high-performance liquid chromatography coupled with a UV/DAD detector (HPLC/UV-DAD). The chromatographic analysis was carried out using a Waters 2695 device (Waters Corporation, Milford, MA, USA) equipped with a UV/DAD detector, auto-injector and column oven. The chromatographic condition was optimized and validated according to Ohmes and Mueller [8]. To do so, the following conditions were used: the mobile phase consisted of acetonitrile—phosphoric acid 0.1% (60:40); an RP-18 Sunfire^®^ column measuring 250 mm × 4.6 mm, 5 µm was used, with a flow rate of 1 mL·min^−1^, injection of 10 µL, detection at 220 nm and column temperature of 30 °C were also used. An RP-18 Sunfire^®^ pre-column was used for validation because such samples, which are derived from soil and plant maceration, are considered unclean [8]. The standard solution was prepared using a SUL standard with a declared content of 99.5%—(Lot 447-89B, Chem Service, West Chester, PA, USA) at a concentration of 0.01 mg·mL^−1^.

This methodology was optimized and validated according to the ICH Q1A R2 (2003) guidebook for the analysis of SUL residues in soils with assessments of the precision, accuracy, linearity, limit of detection and quantification, robustness, specificity and system suitability parameters [11].

*Precision* was assessed by nine determinations, taking into consideration the variation limit of the procedure, *i.e.*, three concentrations of low, medium and high (0.175, 0.250 and 0.325 µg·mL^−1^), and three replicates for each determination. The assays were performed on the same day (repeatability) and on different days (intermediate precision). Precision was expressed as the relative standard deviation (RSD), determined on the same day (repeatability) and on different days (intermediate precision).

*Accuracy* was determined by assessing the rate of recovery of the analyte (sulfentrazone) added to soil that was free of SUL. Nine determinations were carried out, comprising three concentrations of low, medium and high (0.175, 0.250 and 0.325 μg·mL^−1^) and three replicates for each determination. The assays were performed on the same day (intra-day accuracy) and on different days (inter-day accuracy).

*Linearity* was performed in the concentration range from 0.175 to 0.325 µg·mL^−1^, each of which was prepared by adding the standard to the soil and simulating the real analysis conditions in the matrix (soil).

#### 3.1.1. Preparation of the curve with the soil

*Standard Solution A*—SUL (20.0 mg), in triplicate, was weighed on an analytical balance with a precision of 0.01 mg and then quantitatively transferred to a 100 mL volumetric flask and diluted in approximately 50 mL of methanol. Each solution was sonicated for 10 min to ensure solubilization. Then, solvent was added to the final volume, and the solution was homogenized. A diluted solution was subsequently prepared, using a volumetric pipette to transfer 5 mL into a 100-mL volumetric flask; the volume was completed, and the solution was homogenized, leading to a standard solution with a final concentration of 10 µg·mL^−1^.

*Standard Solution B*—Soil free from contamination with the analyte (matrix, 40 g) was weighed in five different 250-mL conical flasks. Next, volumes of 1.4, 1.7, 2.0, 2.3 and 2.6 mL of Standard Solution A were added using a 10-mL calibrated burette. The mixture of Soil + Standard Solution A was left standing until complete solvent evaporation. Then, methanol (80 mL) was added to each conical flask (sample). The solution was stirred for 16 h in a stirrer at 180 rpm. Concentrations of 0.175, 0.213, 0.250, 0.288 and 0.325 µg·mL^−1^, respectively, were obtained. After stirring, the samples were centrifuged for 15 min at 3,200 rpm to achieve separation. Then, a portion of the supernatant was removed with a syringe and filtered through a 0.45-µm PTFE Millipore^®^ membrane into 1.5 mL vials, which were then submitted to the chromatographic analysis.

The standard curve was calculated by plotting the average area values on graphs as functions of concentration. The linear fit equation and the correlation coefficient (r) were determined by the least squares method. The relative standard deviation (RSD) values were also calculated for each point of the curve.

*Limit of Detection (LOD)*: The LOD determination was carried out by considering the signal to be three times greater than the baseline noise. The LOD was determined using the parameters of the analytical curve according to the following Equation (1):

*LOD* = (*SDa x* 3)/*S* (1)

where SDa is the standard deviation of the Y-intercept of at least three calibration curves obtained with analyte concentrations close to the presumed limit of quantification, and S is the slope of the calibration curve.

*Limit of quantification (LOQ)*: The LOQ was determined using the parameters of the analytical curve, according to the Equation (2):

*LOQ* = (*SDa x* 10)/*S* (2)

where SDa is the standard deviation of the Y-intercept of at least three calibration curves obtained with analyte concentrations close to the presumed limit of quantification, and S is the slope of the calibration curve.

*Robustness*: The following factors were considered for the determination of the robustness of the analytical method:

- The variation in the composition of the mobile phase, that is, the ratio of acetonitrile to phosphoric acid 0.1% (65:35), was used as the mobile phase.
- The variation of temperature was based on an oven temperature of 40 °C.
- The variation of the flow rate of the mobile phase was 2 mL·min^−1^.

#### 3.1.2. Specifity and Inherent Stability

To determine the degradation products and evaluate the specificity of the analysis method in soils, SUL was subjected to the following stress conditions.

- Exposure to dry heat: SUL (20 mg) was weighed into a beaker and subjected to a dry heat of 105 °C for 4 h in an oven. After this period, the sample was diluted with methanol to a concentration of 10 mg·mL^−1^.
- Neutral hydrolysis: SUL (20 mg) was weighed and transferred to a 100-mL volumetric flask. Then, ultra-pure water (60 mL) was added, and the mixture was heated in a water bath for 4 h. After that, the sample was diluted with methanol to a concentration of 10 mg·mL^−1^.
- Basic hydrolysis: SUL (20 mg) was weighed and transferred to a 100-mL volumetric flask. Then, sodium hydroxide (60 mL, 0.1 mol·L^−1^) was added, and the mixture was heated in a water bath for 4 hours. After that, the sample was diluted with methanol to a concentration of 10 mg·mL^−1^.
- Acid hydrolysis: SUL (20 mg) was weighed and transferred to a 100-mL volumetric flask. Then, hydrochloric acid (60 mL, 0.1 mol·L^−1^) was added, and the mixture was heated in a water bath for 4 hours. After that, the sample was diluted with methanol to a concentration of 10 mg·mL^−1^.
- Oxidation: SUL (20 mg) was weighed and transferred to a 100-mL volumetric flask. Then, hydrogen peroxide (60 mL, 3%) was added, and the mixture was heated in a water bath for 4 h. After that, the sample was diluted with methanol to a concentration of 10 mg·mL^−1^.
- Exposure to ultraviolet light: SUL (20 mg) was weighed into a 100-mL volumetric flask. The volume was then completed with methanol, and the solution was subjected to exposure, in an ultraviolet radiation chamber, to the energy of a 254-nm lamp. After that, the sample was diluted with methanol to a concentration of 10 mg·mL^−1^.

After the exposure period, samples from each stress condition were taken, placed into HPLC vials and analyzed. The specificity relatively to the soil matrix was also evaluated.

#### 3.1.3. System Suitability

The performance parameters were calculated according to the formulas described below, as described in the literature [12,13,14,15].

*Retention Factor (k′)*: The retention factor was calculated according to the Equation (3):

*k*′ = ((*t* − *to*))/*to* (3)

where t = time (min) measured at the peak maximum, and to = dead time (min).

*Peak Asymmetry (As)*: The peak asymmetry was calculated according to the Equation (4):

*As* = *W*_0.05_/2*f* (4)

where W0.05 = peak width (min) at 5% height, and f = distance (min) between the peak maximum and the main peak end.

*Number of Theoretical Plates (N)*: The number of theoretical plates per column was calculated according to the Equation (5):

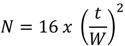
(5)
where t = time (min) measured at the peak maximum, and W = width of the peaks measured at the baseline (min).

*Resolution (Rs)*: The resolution between peaks was calculated according to the Equation (6):

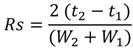
(6)
where t = time (min) measured at the peak maximum, and w = width of the peaks measured at the baseline (min).

### 3.2. Phytoremediation of the SUL Herbicide-containing Soil and Residual Analysis with the Bioindicator

A Haplic Acrisol soil with 120, 104 e 776 g·kg^−1^ of clay, silt and sand, respectively; pH_(H2O)_ = 5.2; MO = 2,0 dag·kg^−1^ was collected in an area with no history of herbicide at a depth of 0.0–0.20 m, and sieved through 4 mm mesh [27]. Soil standard was prepared and then placed in a pot vessel (12 dm^3^), which was covered with a polyethylene film to prevent the loss of herbicide by leaching. Ten kilograms of substrate was used per pot. The pots were irrigated adjusting soil moisture was close to 80% of field capacity, and the herbicide was applied on each individual vessel using a micro-sprayer (glass spray bottle with capacity for 15 mL), under two different doses, namely, 200 and 400 g·ha^−1^ (commercial formulation—Boral^®^). A sample of soil without contamination by SUL was also used for comparison. The experiment randomized design consisted of a 2 (population density) × 3 (dose) factorial with four replications. Two species—wonderbean (*Canavalia ensiformis*) and sunnherb (*Crotalaria juncea*)—were cultivated, where each was chosen for the associated phytoremediation effects. *C. juncea* densities of 0 to 60 plants·m^−2^ and *C. ensiformis* densities of 0 to 10 plants·m^−2^ were used. For both species, the densities correspond to zero, 1× and 2× the recommended amount in the practice of green manuring.

Irrigation was applied three times daily to maintain soil moisture at 60% of field capacity (FC). The FC value was determined in preliminary testing prior to deployment of the experiment, considering a rate of decrease of water content |dθ/dt| = 0.001 d^−1^ [28]. Following this step, the residual evaluation of SUL in the soil was carried out using two techniques for a sensitivity comparison. These approaches are represented by the limit of detection (LOD) and the limit of quantification (LOQ) using the bioindicator *Pennisetum glaucum* (millet) and HPLC.

Fifteen seeds of the bioindicator species were sown into the soil of each pot. After 25 and 42 days, the millet was evaluated according to the plant height, using a graduated scale and the apical meristem as a reference. An analysis of variance was performed, and the mean plant height was compared using the Tukey test at 5% significance.

### 3.3. Residual Evaluation of SUL in the Soil by HPLC

The soil samples obtained after the cultivation of *C. juncea* and *C. ensiformis* were collected in pots at two depths (0.0 to 0.10 m and 0.10 to 0.20 m), following 75 days of phytoremediator sowing. These soil samples were then used for the determination of herbicide persistence in the soil. The chromatographic analysis of the SUL residues was performed with the validated method. The samples were fractioned relative to the depth within the pot to identify whether SUL residues accumulate near the top, close to the root or at the bottom of the pot.

The chromatographic analysis was performed by HPLC using an optimized and validated methodology. The standard solution was prepared from the SUL standard with a declared content of 99.8% and at a concentration of 0.01 mg·mL^−1^.

The SUL residues in the soil were determined by extraction with methanol (80 mL) for each 40 g of soil, where the samples were stirred for 16 h at 180 rotations per min (rpm). After stirring, the samples were centrifuged for 15 min at 3,200 rpm to separate them. Then, a portion of the supernatant was removed with a syringe and filtered through a 0.45 µm PTFE Millipore^®^ membrane into 1.5 mL vials, which were then submitted to chromatographic analysis.

Quantitative analysis was performed by comparing the peak areas of the standard solution obtained from the calibration curve of linearity against those of the sample solutions. The amount of herbicide found in each soil sample was calculated in mg·Kg^−1^ of sample.

## 4. Conclusions

The HPLC-UV/DAD analytical methodology proposed for SUL analysis in this work was optimized and validated. All validation parameters were satisfactory. However, its application must take into consideration the limitations regarding the LOD and LOQ. The bioindicator *P. glaucum* shows a higher sensitivity than the HPLC/UV-DAD technique to sulfentrazone. Any change in SUL concentration caused by the phytoremediation effect of *Canavalia ensiformis* or *Crotalaria juncea* was accurately identified with the bioindicator. However, the bioindicator is not an analytical technique, and there is no true correlation between the size of the species and the analyte content.

The use of chromatography should always be considered for quantitative analysis of the sulfentrazone residue when it is the object of study and must take into account their LD and LQ in performing analysis and confiability of results. Thus, the HPLC-UV/DAD is one more tool, along with the bioindicator, in the evaluation of soils.
